# Successful Sequential Multimodal Therapy for Lingual Rhabdomyosarcoma in a Young Adult: Case Report

**DOI:** 10.1155/crom/7742907

**Published:** 2026-07-21

**Authors:** Jonathan Villanueva Dominguez, Josué Vázquez Arizmendi, Vianey Guadalupe Saldaña Herrera, Ivan Meneses Morales, Gabriel Ernesto Díaz Juarez, Carlos Enrique Arciniega Vega, Rowena Lopez Chávez, Azucena Ocampo Bárcenas

**Affiliations:** ^1^ State Cancer Institute “Dr. Arturo Beltrán Ortega”, Acapulco, Guerrero, Mexico; ^2^ Center for Research on Tropical Diseases, Autonomous University of Guerrero, Acapulco, Guerrero, Mexico, uagro.mx; ^3^ Faculty of Chemical Sciences, Juárez University of the State of Durango, Durango, Durango, Mexico

**Keywords:** adult rhabdomyosarcoma, antiangiogenic therapy, head and neck sarcoma, immunotherapy, lingual rhabdomyosarcoma

## Abstract

Lingual rhabdomyosarcoma (RMS) in adults is an exceedingly rare malignancy, with very few cases reported and no standardized treatment guidelines. We present the case of a 19‐year‐old male diagnosed with lingual RMS who underwent sequential multimodal therapy. The patient initially received doxorubicin combined with ifosfamide chemotherapy followed by cisplatin combined with 5‐fluorouracil, both of which resulted in disease progression. A third‐line regimen with vincristine, actinomycin D, and cyclophosphamide (VAC) together with pembrolizumab achieved only transient stabilization and was limited by toxicity. Subsequent treatment with cetuximab together with bevacizumab induced a significant partial response with acceptable tolerance, which was consolidated with intensity‐modulated radiotherapy (IMRT) to the primary site and cervical lymph nodes. Surveillance imaging confirmed durable locoregional control without evidence of systemic disease, whereas swallowing and phonation were preserved. This case illustrates the value of individualized stepwise management in adult RMS and suggests that the integration of immunotherapy and antiangiogenic therapy may provide meaningful tumor control and functional preservation in selected patients.

## 1. Introduction

Rhabdomyosarcoma (RMS) is the most common pediatric soft‐tissue sarcoma, but it is rare in adults, where outcomes are typically worse [1–3]. In the absence of adult‐specific treatment guidelines, pediatric regimens such as VAC (vincristine, actinomycin D, cyclophosphamide) are often extrapolated, though with modest benefit [4, 5]. Lingual RMS in young adults is exceptionally rare, with only a handful of cases documented. We describe a young adult with lingual RMS successfully managed with a stepwise multimodal strategy, highlighting the feasibility of integrating conventional and emerging therapies.

## 2. Case Presentation

A previously healthy 19‐year‐old male presented in April 2024 with nocturnal dysphonia and sialorrhea. Physical examination revealed a tongue lesion, initially treated with dexamethasone without improvement. Over the following weeks, the patient developed progressive dysphagia and rapid tumor growth, prompting excisional biopsy.

**Figure 1:**
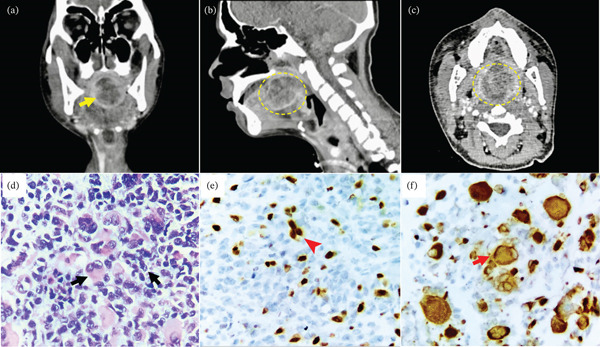
Lingual rhabdomyosarcoma: imaging, histopathologic, and immunohistochemical evaluation. (a–c) Contrast‐enhanced computed tomography in coronal, sagittal, and axial planes at diagnosis, demonstrating a well‐defined enhancing soft tissue mass involving the mid and posterior thirds of the tongue (arrows/circle), without evidence of adjacent bone infiltration. (d) Histopathologic examination showing primitive mesenchymal cells and rhabdomyoblast‐like cells with eosinophilic cytoplasm at different stages of myogenic differentiation (H&E, 40x). (e) Nuclear myogenin expression in neoplastic cells, supporting skeletal muscle differentiation (40x). (f) Cytoplasmic desmin positivity in tumor cells, consistent with myogenic differentiation (40x).

Histopathology revealed a malignant mesenchymal neoplasm consistent with RMS. Neck CT scan showed a solid tumor measuring 5.2 × 3.9 cm involving the base and mobile portion of the tongue, with cervical lymphadenopathy (Figure 1a–c). Histologic review confirmed proliferation of primitive mesenchymal cells at various stages of myogenesis; immunohistochemistry demonstrated nuclear myogenin expression and cytoplasmic desmin positivity (Figure 1d–f).

The case was discussed in a multidisciplinary tumor board, including medical oncology, surgical oncology, radiation oncology, radiology, and pathology. Given the tumor location and the potential functional morbidity associated with radical surgery, a sequential systemic treatment strategy was recommended. Treatment was initiated with anthracycline‐ifosfamide chemotherapy based on the sarcomatous histology. The patient received three cycles of doxorubicin + ifosfamide (75 mg/m^2^ of doxorubicin and 9 g/m^2^ of ifosfamide divided over 3 days), but local progression was observed. Given this lack of response, PD‐L1 testing was requested to explore potential eligibility for immunotherapy, based on treatment paradigms in head and neck malignancies. The PD‐L1 (SP‐142) result was negative (CPS 0).

Management was then adapted to a cisplatin + 5 − fluorouracil (5‐FU) regimen (cisplatin 100 mg/m^2^ plus 5‐FU 1000 mg/m^2^/day for 96 h), as used in squamous cell tumors of the head and neck. However, after two cycles, clinical progression was evident. In light of this, a third‐line regimen with VAC (vincristine 1.5 mg/m^2^, actinomycin D 0.045 mg/kg, and cyclophosphamide 1.2 mg/m^2^) was initiated, based on case reports of similar histologies in pediatric tumors [6, 7]. Pembrolizumab 200 mg every 3 weeks was added, drawing on the findings of KEYNOTE‐048 regarding PD‐L1 independent overall survival benefit when combined with chemotherapy [8].

The patient received three cycles, achieving stable disease according to RECIST 1.1 criteria, without meaningful tumor shrinkage, and experienced considerable toxicity according to CTCAE v5.0 criteria (Grade 3 neutropenia, Grade 2 mucositis, and Grade 2 nausea/vomiting). Given the absence of additional standard options, treatment was switched to cetuximab + bevacizumab, supported by Phase I/II studies in refractory head and neck tumors [9, 10]. After six cycles of cetuximab + bevacizumab, a partial response according to RECIST 1.1 criteria was observed, with a reduction in the longest tumor diameter from 5.2 cm at baseline to approximately 3.6 cm on follow‐up imaging (≈30% decrease), with only mild toxicity (Grade 1 skin rash and Grade 1 fatigue). CT imaging confirmed partial response, with no evidence of disease in the lung, bone, or brain. Local control with surgery was proposed, but the patient declined due to concerns about functional sequelae.

Intensity‐modulated radiotherapy (IMRT) was administered to the primary site and cervical lymph nodes, initially at 2 Gy per fraction and later reduced to 1.5 Gy per fraction due to mucosal toxicity. The patient developed xerostomia and odynophagia but achieved significant local tumor control. Maintenance therapy with cetuximab and bevacizumab was delivered in six additional cycles administered every 3 weeks.

**Figure 2:**
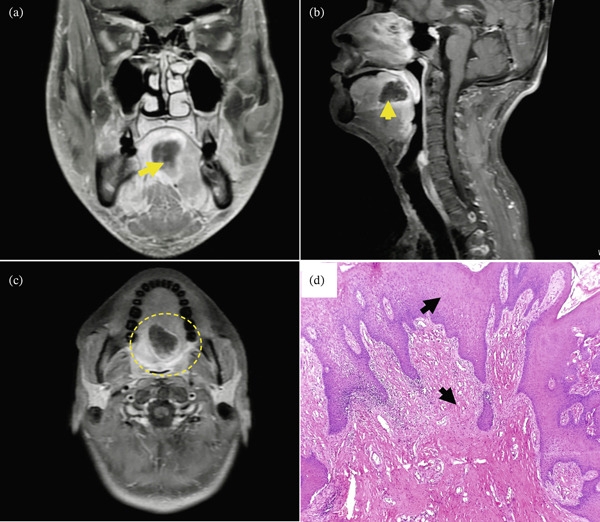
Lingual rhabdomyosarcoma: magnetic resonance imaging and posttreatment histopathologic correlation. (a–c) Posttreatment contrast‐enhanced T1‐weighted magnetic resonance imaging with fat suppression demonstrating residual posttherapeutic enhancing tissue extending toward the right lateral margin of the tongue (arrows/circle). No cervical lymphadenopathy or adjacent bone or salivary gland involvement was identified. The lesion measured approximately 40 × 28 × 32 mm. (d) Posttreatment biopsy showing fibrosis and chronic inflammatory changes without evidence of viable neoplastic cells (H&E, 10x).

**Figure 3:**
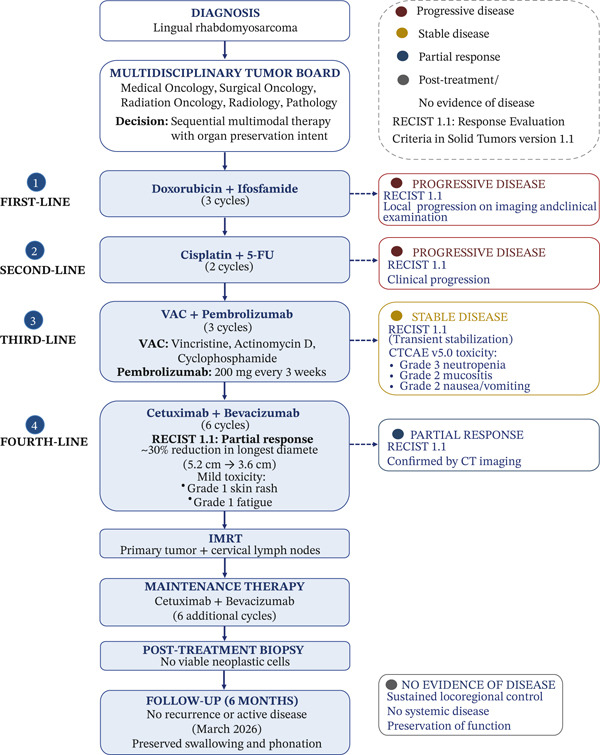
Sequential multimodal treatment strategy and therapeutic responses in lingual rhabdomyosarcoma. Progressive disease was observed after doxorubicin plus ifosfamide and cisplatin plus 5‐FU. VAC plus pembrolizumab achieved transient stable disease, whereas cetuximab plus bevacizumab induced partial response according to RECIST 1.1 criteria. IMRT and maintenance therapy were subsequently administered. Posttreatment biopsy showed no viable neoplastic cells, and follow‐up demonstrated no evidence of recurrence or active disease.

A fluorodeoxyglucose positron emission tomography/computed tomography (FDG‐PET/CT) revealed a lesion in the left half of the tongue extending beyond the lingual septum, hypodense in the noncontrast phase with peripheral enhancement, measuring approximately 36 × 30 mm and maximum standardized uptake value (SUVmax) 1.4. Irregular margins were noted on the anterior tongue with uptake (SUVmax 4.0). Cervical lymph nodes at Levels II A/B and V bilaterally measured up to 6 mm (SUVmax 1.5). Mild esophageal uptake (SUVmax 2.1) and diffuse gastric uptake related to gastrostomy were also observed. Surgery for local control was again offered given the favorable response, but the patient declined. Posttreatment magnetic resonance imaging confirmed these findings (Figure 2a–c). Instead, an excisional biopsy was performed, showing reactive squamous epithelium and chronic subepithelial inflammation (Figure 2d). A schematic overview of the sequential therapeutic strategy and clinical responses is presented in Figure 3.

**Figure 4:**
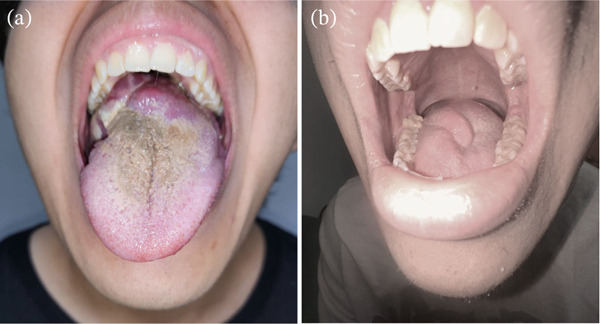
Clinical presentation of lingual rhabdomyosarcoma. (a) Initial appearance showing an infiltrating mass in the tongue. (b) Posttreatment resolution with preservation of tongue morphology following multimodal therapy.

Surveillance began in February 2025. Six months later (September 2025), CT imaging showed no evidence of tumor activity in lymph nodes, lungs, or bone. At the last follow‐up (March 2026), the patient remained without evidence of recurrence or active disease, with preserved swallowing, phonation, and quality of life. Continued follow‐up with contrast‐enhanced CT imaging of the neck, thorax, and abdomen was scheduled as part of ongoing monitoring (Figure 4a,b). Previously reported cases of lingual rhabdomyosarcoma in adults have primarily relied on surgical resection combined with radiotherapy or chemotherapy, often with significant functional morbidity. In contrast, our case highlights a nonsurgical, organ‐preserving strategy based on sequential systemic therapy and radiotherapy, achieving sustained local tumor control while preserving swallowing and phonation. A comparison of previously reported cases and their therapeutic approaches is summarized in Table 1. This comparison underscores the potential value of individualized multimodal approaches in selected patients.

**Table 1:** Clinicopathological features and outcomes of reported lingual rhabdomyosarcoma cases.

Study	Age/sex	Primary site	Histologic subtype	Treatment	Outcome/follow‐up
Díez‐Montiel et al. 2023 [1]	46/M	Dorsum of tongue	Embryonal RMS with fusocellular areas	Excisional biopsy → partial glossectomy + buccinator flap reconstruction → eight cycles VAC	Disease‐free with preserved tongue function at 42 months
Yang et al. 2023 [2] (Case 1)	4‐month‐old/F	Left lingual margin	Spindle cell RMS	Surgery → 4 VAC + 4 VA	Complete remission at 26 months
Yang et al. 2023 [2] (Case 2)	3‐month‐old/F	Base of tongue	Spindle cell RMS	Surgery → VAC/VA → relapse → chemotherapy → surgery → proton RT	Complete remission at 31 months
Wagemans et al. 2010 [4]	19–41 years (Seven adults)	Head and neck (mixed sites; not isolated tongue)	Embryonal RMS	Multimodal therapy (VIA/VIP ± surgery ± RT)	Three of seven disease‐free; median follow‐up 4.5 years
**Current case**	**19/M**	**Tongue**	**Rhabdomyosarcoma**	**AI → Cisplatin/5-FU → VAC + pembrolizumab → cetuximab + bevacizumab → IMRT**	**Durable local control; preserved swallowing and phonation**

*Note:* Values shown in bold correspond to the current case.

Abbreviations: 5‐FU, 5‐fluorouracil; AI, doxorubicin plus ifosfamide; IMRT, intensity‐modulated radiotherapy; RMS, rhabdomyosarcoma; RT, radiotherapy; VA, vincristine, actinomycin D; VAC, vincristine, actinomycin D, cyclophosphamide.

## 3. Discussion

Lingual RMS in adults is an exceptionally rare entity, representing less than 1% of head and neck sarcomas, with very limited therapeutic evidence available [1, 11–14]. Documenting individual cases therefore contributes valuable insights into clinical decision making.

In our patient, first‐line treatment with anthracycline, ifosfamide, and second‐line cisplatin + 5 − FU were both ineffective, which is consistent with prior reports showing poor responses in adult RMS compared with pediatric populations [11–15]. Pediatric backbones such as VAC remain standard in children but generally yield modest results in adults [5–7]. In this case, VAC provided only transient stabilization, underscoring the need for alternative strategies.

Given the lack of durable responses, pembrolizumab was introduced in combination with VAC. This decision was guided by findings from KEYNOTE‐048 [8], where PD‐L1 independent overall survival benefit was reported in head and neck squamous cell carcinoma, and by the SARC028 trial, which provided the first prospective evidence of pembrolizumab activity in sarcomas [16], albeit with modest efficacy. Case reports have also described temporary disease control with pembrolizumab plus chemotherapy in pleomorphic RMS [17]. In our patient, pembrolizumab plus VAC produced transient stabilization but no objective response, reflecting the limited single‐agent efficacy of immune checkpoint inhibitors in unselected sarcomas [18]. The limited response observed in our patient may be explained by the absence of PD‐L1 expression and the intrinsically low immunogenicity of most soft‐tissue sarcomas, which are typically characterized by low tumor mutational burden and an immunologically “cold” tumor microenvironment.

The most notable response occurred with cetuximab plus bevacizumab. Although scarcely reported in RMS, this combination has demonstrated activity in Phase I/II studies of refractory head and neck tumors [9, 10]. VEGF inhibition by bevacizumab may normalize RMS vasculature and enhance therapeutic response, whereas EGFR blockade by cetuximab has shown synergy in epithelial tumors [19, 20]. Although EGFR expression was not assessed in this case, previous studies have demonstrated variable EGFR expression in RMS, suggesting a possible therapeutic vulnerability [21]. Furthermore, VEGF‐driven angiogenesis is a well‐recognized feature of sarcoma biology, and VEGF inhibition may contribute to vascular normalization, enhanced drug delivery, and increased radiosensitivity [20]. In this context, the clinical response observed with cetuximab plus bevacizumab may reflect a potential synergistic interaction targeting both tumor cell signaling and the tumor microenvironment. Nevertheless, in the absence of molecular or biomarker confirmation, these findings should be interpreted cautiously and considered hypothesis‐generating rather than definitive evidence of mechanism. In our case, six cycles of this regimen induced a significant partial remission with acceptable toxicity, which was further consolidated by IMRT. Although follow‐up remains relatively limited, the patient has maintained locoregional disease control without evidence of recurrence or active tumor activity as of March 2026, supporting the potential durability of this multimodal strategy.

This therapeutic sequence aligns with emerging evidence suggesting that combining ICIs with antiangiogenic agents or chemotherapy can improve outcomes in advanced soft‐tissue sarcomas. For instance, the PEMBROSARC program reported a 30% response rate in sarcomas with tertiary lymphoid structures [22], whereas the NiTraSarc Phase II trial (nivolumab plus trabectedin) showed disease stabilization in a subset of patients [23]. Other early‐phase studies (e.g., trabectedin + avelumab or durvalumab [24, 25]; sintilimab + doxorubicin/ifosfamide [26] support the rationale for early integration of novel combinations.

Despite these advances, immune checkpoint inhibitor monotherapy continues to show limited efficacy in most soft‐tissue sarcomas, with overall response rates of 10%–15% [18, 24, 25]. This has reinforced interest in combination regimens, particularly ICI plus tyrosine kinase inhibitors (TKIs), which appear more promising in early trials [23, 27–29].

Comprehensive molecular characterization, including FOXO1 fusion testing, broad genomic profiling, and immunohistochemical assessment of EGFR/VEGF expression, was not available during the patient′s treatment course. Access to these assays remains limited in many resource‐constrained oncology settings, including our institution, which restricted deeper biological interpretation of therapeutic response. Although EGFR testing by FISH or PCR was explored, these approaches were not routinely accessible or clinically validated in this context. Therefore, treatment decisions were guided by clinicopathologic findings, multidisciplinary discussion, biological plausibility, and available evidence from early‐phase sarcoma and head and neck cancer studies.

Although this case does not establish a novel biological mechanism or predictive biomarker, it provides clinically relevant insight into treatment sequencing in a rare and therapeutically challenging scenario. The observed response to cetuximab plus bevacizumab, following multiple prior lines of therapy, suggests that alternative pathway targeting may be beneficial even in tumors not traditionally considered for such approaches. What distinguishes our case is not only the rarity of lingual RMS in a young adult, but also the feasible integration of immunotherapy and antiangiogenic therapy, achieving durable partial remission and preservation of swallowing and phonation. This outcome provides real‐world evidence that novel therapeutic combinations, even when supported only by early‐phase data, may offer clinically meaningful benefit in highly refractory tumors. Longer surveillance will be necessary to determine the durability of disease control.

## 4. Conclusions

Adult lingual RMS is an exceptionally rare and challenging malignancy for which no standardized treatment guidelines exist. Our report contributes to the scarce existing literature and underscores the clinical importance of documenting such cases. A flexible, sequential multimodal strategy integrating chemotherapy, radiotherapy, immunotherapy, and antiangiogenic therapy achieved sustained locoregional tumor control without evidence of active disease at the latest follow‐up and preserved vital functions. The strength of this case lies in tailoring each treatment step to tumor response and patient tolerance, illustrating the value of individualized management in adult RMS. The outcome supports the early incorporation of emerging agents in the management of rare sarcomas. Moreover, the combination of immune checkpoint inhibitors and antiangiogenic agents proved feasible and effective in this context, although scarcely reported. Future collaborative research should focus on biomarker discovery and validation of sequencing strategies to optimize outcomes in this underexplored population.

## Author Contributions

All authors contributed to the conceptualization, writing, and critical review of this case report.

## Funding

No specific funding was received for this work.

## Consent

Written consent for publication was obtained from the patient.

## Conflicts of Interest

The authors declare no conflicts of interest.

## Data Availability

The data that support the findings of this study are available on request from the corresponding author. The data are not publicly available due to privacy or ethical restrictions.
